# Introduction of artificial light at night increases the abundance of predators, scavengers, and parasites in arthropod communities

**DOI:** 10.1016/j.isci.2023.106203

**Published:** 2023-02-14

**Authors:** Jeffrey A. Brown, Julie L. Lockwood, Max R. Piana, Caroline Beardsley

**Affiliations:** 1 Department of Biology, La Salle University, Philadelphia, PA, USA; 2 Department of Ecology, Evolution, and Natural Resources, Rutgers University, New Brunswick, NJ, USA; 3 Northern Research Station, USDA Forest Service, Amherst, MA, USA; 4 Research Assistant Program, Aresty Research Center, Rutgers University, New Brunswick, NJ, USA

**Keywords:** Environmental science, Ecology, Entomology

## Abstract

While recent studies explore the negative impacts of light pollution on arthropods, few studies investigated community-level responses to artificial light. Using an array of landscaping lights and pitfall traps, we track community composition over 15 consecutive days and nights, including a five-night pre-light period, a five-night during-light period, and a five-night post-light period. Our results highlight a trophic-level response to artificial nighttime lighting with shifts in the presence and abundance of predators, scavengers, parasites, and herbivores. We show that associated trophic shifts occurred immediately upon the introduction of artificial light at night and are limited to nocturnal communities. Lastly, trophic levels reverted to their pre-light state, suggesting many short-term changes in communities are likely the result of behavioral shifts. These trophic shifts may become common as light pollution increases, implicating artificial light as a cause of global arthropod community change and highlighting light pollution’s role in global herbivorous arthropod decline.

## Highlights

• Introducing light pollution immediately shifts nocturnal arthropod communities
• Artificial light increases the abundance of predators in arthropod communities
• Community abundance increases with the introduction of light
• Arthropod communities revert to pre-light composition once the light is removed

## Graphical abstract

**Figure:**
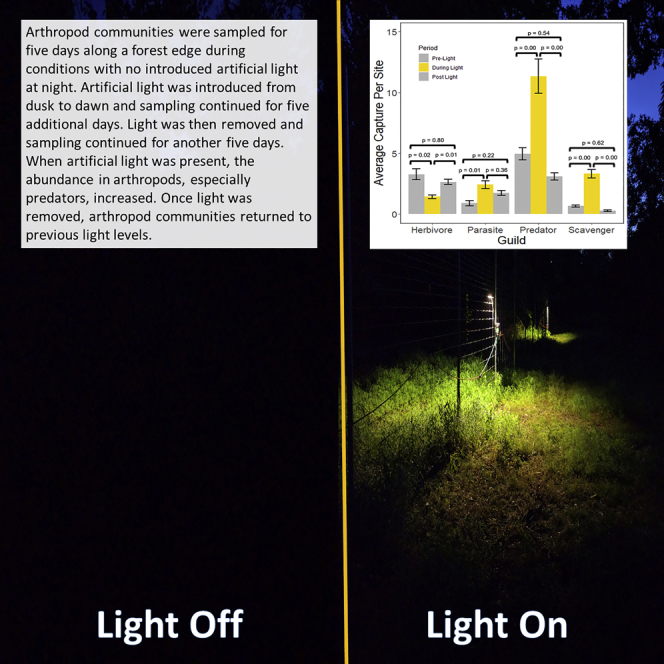

## Introduction

The Anthropocene is defined by high levels of human influence, resulting in atmospheric, geologic, hydrologic, and biospheric shifts.^1^ These shifts are often linked to the increasing human population and have been highlighted by researchers over the last three decades.^2^^,^^3^^,^^4^ Increases in population and urbanization, along with the invention and adoption of new technologies such as light-emitting diodes (LEDs), have disturbed light-dark cycles on a global scale.^5^^,^^6^ Currently, artificial light is a common part of anthropogenic nightscapes, with many areas, especially cities, regularly brighter than nights during the full moon.^7^ Artificially bright nights have been identified as a critical and growing threat to biodiversity; each year an additional 6% of the total land is influenced by nighttime light pollution.^8^^,^^9^^,^^10^ With most of the world impacted by artificial light at night (ALAN) and more areas affected each year, understanding how artificial light changes and shapes ecological communities is critical to future conservation, management, and city planning practices.

Light levels influence a wide range of species’ behavior, including reproduction, migration, foraging, predation, signaling, and other behavioral patterns.^11^^,^^12^^,^^13^^,^^14^ As such, the alteration of natural light patterns can negatively impact wildlife and poses a threat to biodiversity.^7^^,^^9^^,^^15^^,^^16^ However, the way in which artificial light alters natural behaviors has only recently been explored experimentally (see Refs. 17,18,19,20 Many artificial light researchers used urban-rural gradients to compare community composition and species abundance in areas of high ALAN to areas without ALAN^5^). Other studies have compared individual behaviors seen around artificial lights to behaviors seen in non-light-disturbed environments.^21^^,^^22^^,^^23^^,^^24^ One of the most striking conclusions from these studies is that predatory species are disproportionally attracted to artificial light at night compared to other trophic levels. The resulting increase in predator abundance around lights has been known as the *night-light niche*.^25^^,^^26^^,^^27^ The night-light niche influences both diurnal predators, which increase their activity patterns during typically dark periods, as well as nocturnal visual predators, which typically alter their hunting patterns in response to changes in natural light levels such as cycles of the moon.^28^^,^^29^ In cases in which urban-rural gradients are used to look for evidence of night-light niche, increased predation may instead be responding to underlying changes in urban environments or supplemental food sources that are unrelated to increased artificial light.^30^^,^^31^ Additionally, because these studies compare communities impacted and unaffected by ALAN, they lack comparisons to pre-light communities; thus, it is harder to separate potential trophic differences stemming from urban-rural landscape dissimilarities and the introduction of artificial light.^32^^,^^33^^,^^34^

Using an array of pitfall traps and artificial lights, we seek to document the extent and temporal speed of artificial-light-induced community-level shifts by experimentally investigating how naive forest arthropod communities (i.e., a community that has not previously experienced light pollution) respond to the introduction of ALAN. Through experimental manipulation, we established ALAN as a mechanistic driver in community change by monitoring compositional change before and after the introduction of artificial light while including a control group that was exposed to only ambient light over the study period. We continued to sample after the removal of ALAN to investigate if these communities returned to their pre-disturbed state or if artificial light impacts persisted even when lights are turned off or had been permanently removed (see Davies et al.^22^). Our results provide insights into how arthropod communities will respond as light pollution spreads to non-light-polluted environments. Additionally, we gain insights into the speed at which communities respond to changes in levels of ALAN.

## Results

**Figure 1:**
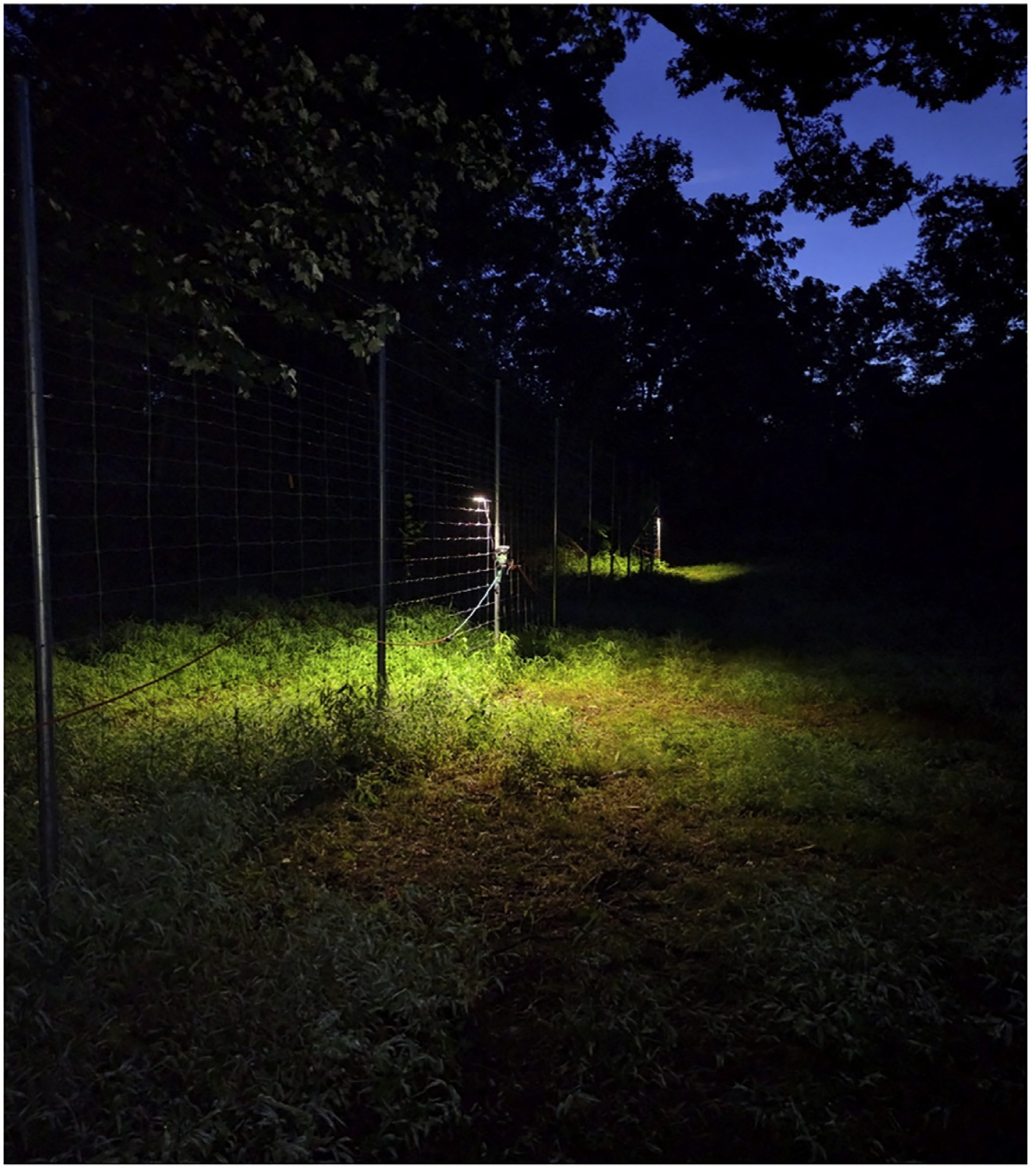
A photo of the experimental design showing two of the light installations The photo was taken 15 min prior to dawn. Photo by Jeff Brown.

All traps spent most of the diurnal periods experiencing light levels of ∼100,000 lux (i.e., full daylight) although traps did experience light levels that dipped to 2,000–10,000 lux as cloud cover shifted during the day (between sunrise and sunset). Light levels at night (i.e., after civilian dusk) ranged from 700 lux to 0.00 lux for control traps and during the pre- and post-light time periods. Light levels steadily decreased from civilian dusk until an hour after dusk after which traps steadied at levels less than 1 lux (average 0.03 lux, SD 0.01 lux, with a peak of 0.06 lux) during the full moon (on August 18^th^). The light level at night over traps during the night when the light was on was significantly higher (t-test, p < 0.05, Figure 1) than ambient nighttime light levels with an average light level of 10.31 lux (SD 1.12 lux). Light levels over control traps never exceeded ambient night light levels (t-test, p < 0.05), and the ambient night light level was consistent during pre-light, during-light, and post-light time periods (ANOVA, p < 0.05).

**Figure 2:**
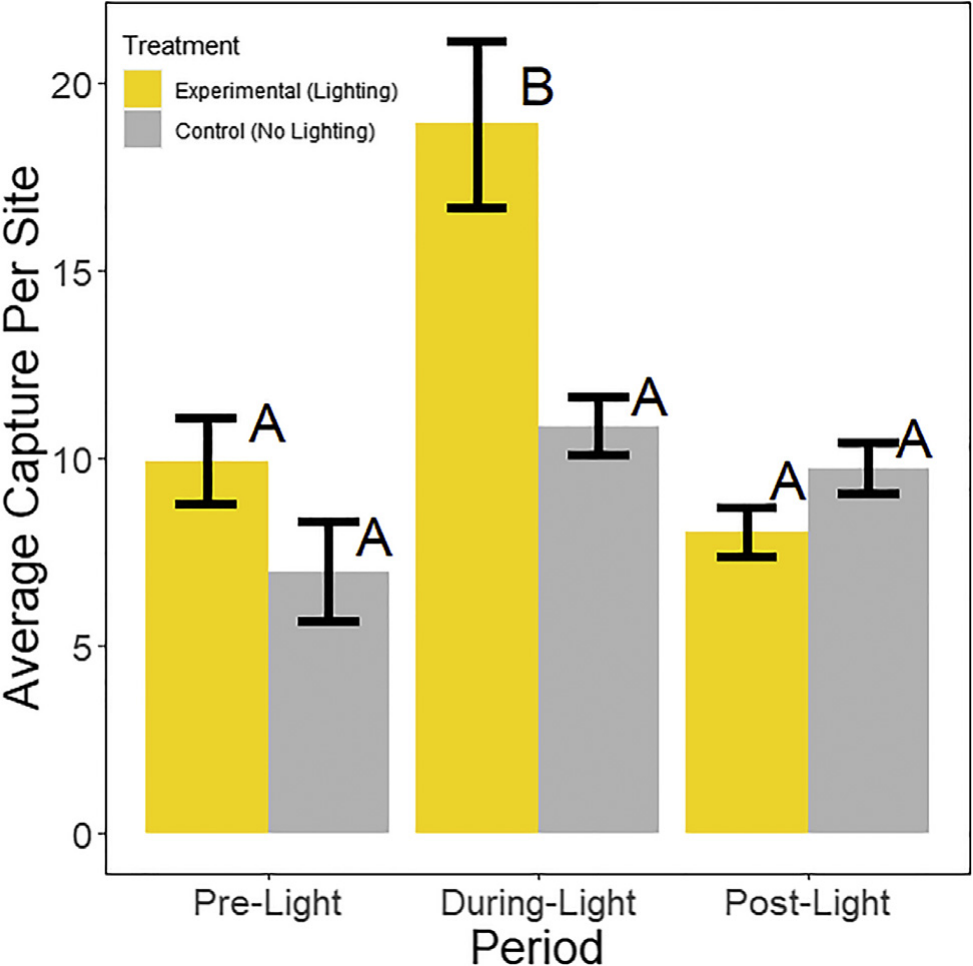
Average abundance per trap for the three time periods during the night for both the experimental and control plots During all three time periods, the control showed no significant change in abundance and was also not significantly different than the pre-light or post-light treatment groups. The only difference in the number of arthropods captured was seen during the treatment group when lights were on. Letters indicate groupings at a significance level of p < 0.05. Error bars show SE around the mean. See Table 1 for specific values.

Over the course of this experiment, we captured 6,594 individual arthropods representing 11 classes, 39 orders, and 145 families. We exclude members of Collembola from this count as they could not accurately be identified below the family level. Average per-site abundance did not change during any dawn-to-dusk period over the course of the experiment. Additionally, per-order abundance did not change during the day (dawn to dusk) over the course of the experiment. The average abundance per night (dusk to dawn) was consistent across control traps during all time periods (Figure 2 and Tables 1 and 2). Additionally, the abundance in experimental traps did not differ from control traps and did not differ between the pre- and post-light periods (Figure 2, Tables 1 and 2). However, abundance in the experimental traps during the light period was significantly higher than both the pre- and post-light levels as well as higher than the control traps during the same time period (Tables 1 and 2, and Figure 2).

**Table 1:** Average abundance per site across all guilds during the three time periods at night for experimental and control sites

Period	Treatment	Average captures per site	SE
Pre	Light	9.94	0.58
During	Light	18.92	1.12
Post	Light	8.06	0.33
Pre	Control	7.00	1.60
During	Control	10.88	0.89
Post	Control	9.76	0.64

**Table 2:** Results from Tukey’s HSD comparing average abundance per site during each period at night for both experimental and control sites

	Pre-light experimental	During-light experimental	Post-light experimental	Pre-light control	During-light control	Post-light control
Pre-light experimental	0 (0 | 0)	−8.98 (−14.86 | −3.10) ∗	1.88 (−4.00 | 7.76)	2.94 (−4.26 | 10.14)	−0.94 (−8.14 | 6.26)	0.18 (7.02 | −7.38)
During-light experimental	8.98 (3.10 | 14.86) ∗	0 (0 | 0)	10.86 (4.98 | 16.74) ∗	11.92 (4.72 | 19.12) ∗	8.04 (2.84 | 12.24) ∗	9.16 (1.96 | 16.36) ∗
Post-light experimental	−1.88 (−7.76 | −4.00)	−10.86 (−16.74 | −4.98) ∗	0 (0 | 0)	1.06 (−6.14 | 8.26)	−2.82 (−10.02 | 4.38)	−1.70 (−8.90 | 5.50)
Pre-light control	−2.94 (−10.14 | 4.26)	−11.92 (−19.12 | −4.72)∗	−1.06 (−8.26 | 6.14)	0 (0 | 0)	−3.88 (−12.20 | 4.43)	−2.76 (−11.07 | 5.55)
During-light control	0.94 (−6.26 | 8.14)	−8.04 (−12.24 | −2.84) ∗	2.82 (−4.38 | 10.02)	3.88 (−4.43 | 12.20)	0 (0 | 0)	1.12 (−7.20 | 9.44)
Post-light control	−0.18 (−7.38 | 7.02)	−9.16 (−16.36 |-1.96) ∗	1.70 (−5.50 | 8.90)	2.76 (−5.55 | 11.07)	−1.12 (−9.44 | 7.20)	0 (0 | 0)

Differences in average abundance between sites (row minus column) are shown with upper and lower 95% confidence intervals. Asterisks next to values indicate a significant difference between the two groups at a level of p < 0.05.

**Figure 3:**
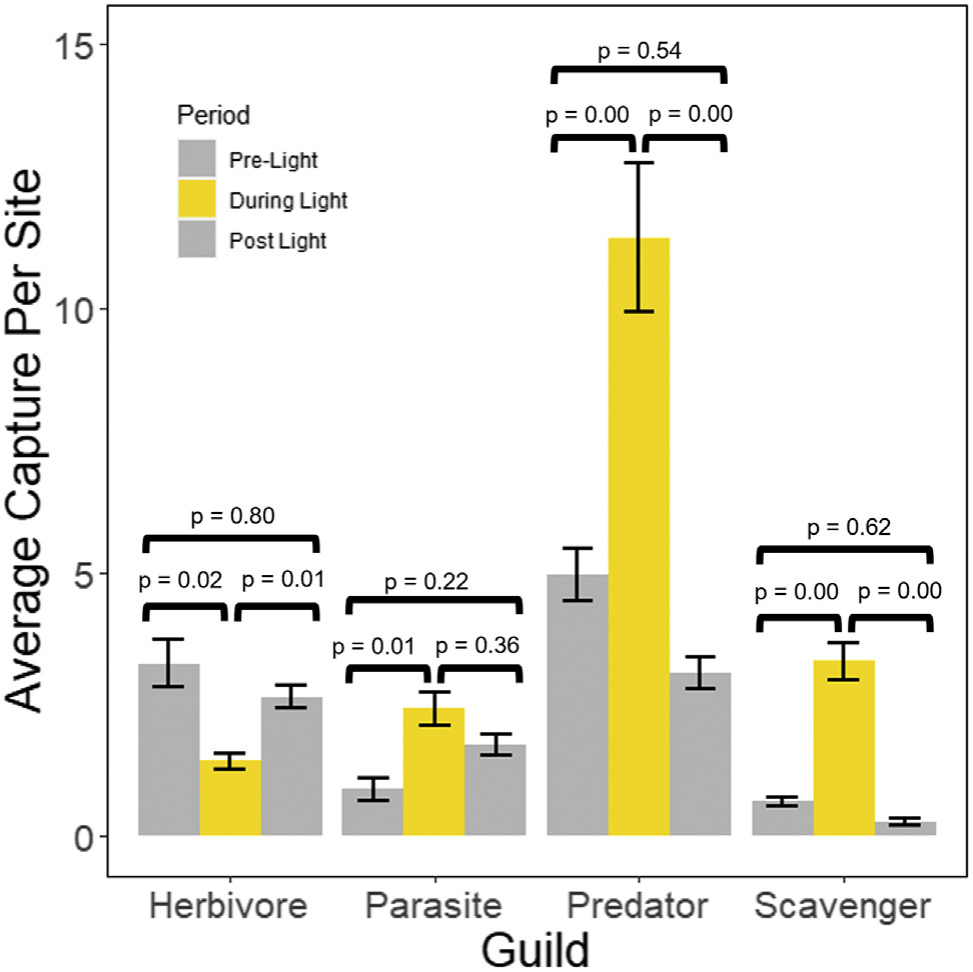
Average abundance caught across experimental traps per night during the three time periods separated by functional guild Significant differences are seen in all guilds when the light is on compared to before the light was turned on. Both herbivores and parasites do not return to their pre-light exposure abundances after light is removed. Herbivore populations are reduced in post-light levels compared to pre-light levels, and parasite populations are elevated. P-values from Tukey’s HSD comparison are shown for each guild. Error bars show SE around the mean.

No changes in community composition were observed in diurnal communities over the course of the experiment. However, nocturnal communities experienced significant changes, with the four most abundant trophic guilds undergoing significant shifts in abundance when the light was on (Figure 3). Increases in the average nightly abundance of parasites, predators, and scavengers were evident when compared to pre-light and post-light conditions (Table 3 and Figure 3). A decrease in guild abundance was seen in herbivore abundance when the light was present compared to pre- and post-light levels (Figure 3 and Table 3).

**Table 3:** Average captures per site for each guild during the pre-, during-, and post-light treatment for experimental sites at night

Guild	Average captures per site & (SE)			Difference between averages & (95% confidence intervals)		
	Pre	During	Post	Pre-during	During-post	Pre-post
Detritivore	0.14 (0.04)	0.14 (0.04)	0.04 (0.01)	0.00 (−0.16 | 0.16)	0.06 (−0.10 | 0.23)	0.06 (−0.10 | 0.23)
Herbivore	3.28 (0.19)	1.45 (0.09)	2.65 (0.04)	1.83 (0.43 | 3.23) ∗	−0.82 (−1.57 | −0.07) ∗	0.63 (0.04 | 1.22) ∗
Parasite	0.90 (0.15)	2.42 (0.21)	1.74 (0.13)	−1.52 (−2.71 | −0.33) ∗	0.68 (−0.51 | 1.87)	−0.84 (−2.03 | 0.35)
Predator	4.96 (0.34)	11.34 (0.94)	3.10 (0.19)	−6.38 (−10.56 | −2.20) ∗	8.24 (4.06 | 12.42) ∗	1.86 (−2.32 | 6.04)
Scavenger	0.66 (0.06)	3.32 (0.22)	0.28 (0.04)	−2.66 (−3.63 | −1.70) ∗	3.04 (2.07 | 4.01) ∗	0.38 (−0.60 | 1.35)

Averages are presented with standard errors in parentheses. Differences between averages, as calculated by Tukey’s HSD, are also shown for each comparison with upper and lower 95% confidence intervals. Asterisks indicate significant differences at a level of p < 0.05.

**Figure 4:**
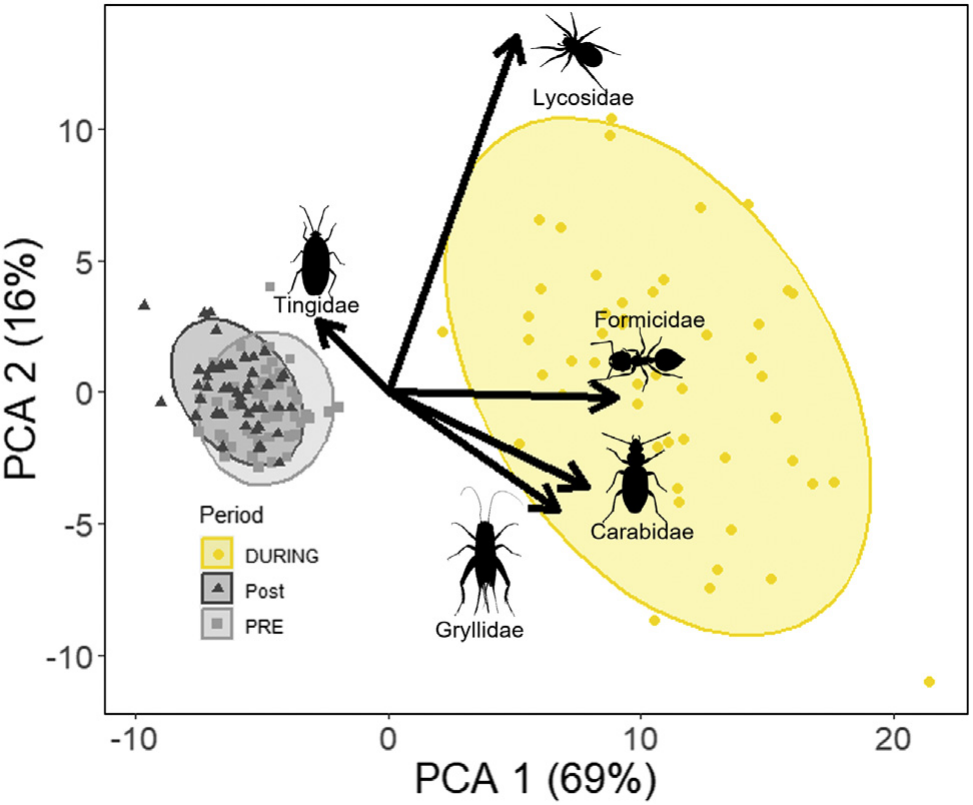
Results of a principal component analysis with the communities from each night of the experiment Each dot represents one night during the experiment. Clusters are set around each time period, and no significant difference is seen between the pre- and post-light clusters, but the during-light cluster is significantly different than the other groups. Families with a weighting under −0.1 or over 0.1 on either PC1 or PC2 are displayed with arrows and visual representation.

Our principal component analysis captured 85% of the total variance in community composition using the first two components (Figure 4). We show a significant difference between pre-light and during-light community compositions as well as during-light and post-light communities (Table S1. Results of pairwise PERMANOVA, related to Figure 4). This shift in composition was seen immediately as the assemblage of arthropod species collected on the first night post-light exposure was more similar in composition to all other light-influenced communities than it was to the pre-light community (pvclust. bootstrap probability 100). Additionally, the during-light community composition had larger dispersion, indicating higher compositional variability between nights and trap locations than those of both the pre-light and post-light communities (betadisper, Table S2. Results of Tukey HSD for dispersion, related to Figure 4). We observed no significant difference between the pre-light and post-light communities in composition or dispersion. Additionally, we saw no shift in the control communities over the course of the study, and the control communities did not significantly differ from the pre- and post-light communities (Table S2. Results of Tukey HSD for dispersion, related to Figure 4). Five families were identified as contributing a weight greater than 0.1 on either PC1 or PC2 and thus likely influenced the differences between the overall communities. These families, in order of contribution, were Lycosidae (wolf spiders), Formicidae (ants), Carabidae (ground beetles), Gryllidae (crickets), and Tingidae (lace bugs).

**Figure 5:**
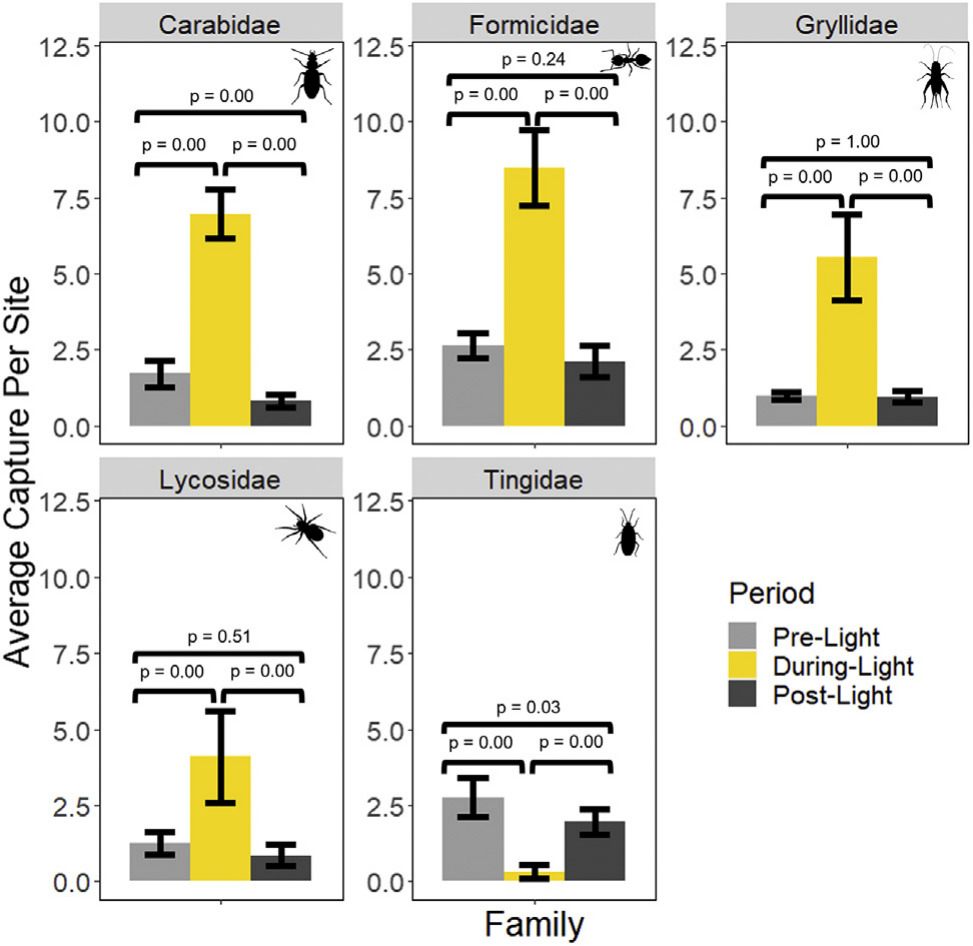
Shifts in key families identified by the weighting of the PCA Significant differences from the light group are seen in Carabidae, Formicidae, Gryliidae, Lycosidae, and Tingidae. P-values from Tukey’s HSD comparison are shown for each light condition. Error bars show SE around the mean.

We found that all five families changed in composition when the light was on as compared to when it was off. Carabidae, Gryllidae, Formicidae, and Lycosidae all increased in abundance when the light was on but returned to pre-light levels once the light was off (Figure 5 and Table 4), and Tingidae showed a reduction in abundance when the light was turned on followed by an increase in abundance when the light was turned off. However, this increase in abundance did not return the Tingidae abundance to pre-light levels (Figure 5 and Table 4).

**Table 4:** Average captures per site for each focal family (PCA weighting above + or – 0.1 on either PCA 1 or PCA 2) during the pre-, during-, and post-light treatment for experimental sites at night

Family	Average captures per site & (SE)			Difference between averages & (95% confidence intervals)		
	Pre	During	Post	Pre-during	During-post	Pre-post
Carabidae	1.72 (0.23)	6.96 (0.41)	0.82 (0.10)	−5.24 (−5.74 | −4.73) ∗	6.14 (5.03 | 7.24) ∗	0.90 (0.19 | 1.61) ∗
Formicidae	2.64 (0.21)	8.48 (0.62)	2.12 (0.26)	−5.84 (−6.59 | −5.09) ∗	6.63 (5.61 | 7.11) ∗	0.52 (−0.23 | 1.27)
Gryllidae	0.98 (0.07)	5.54 (0.73)	0.96 (0.09)	−4.56 (−5.34 | −3.78) ∗	4.58 (3.80 | 5.35) ∗	0.02 (−0.96 | 1.00)
Lycosidae	1.26 (0.29)	4.10 (0.77)	0.86 (0.18)	−2.84 (−3.70| −1.98) ∗	3.24 (2.38 | 4.10)	0.40 (−0.46 | 1.26)
Tingidae	2.76 (0.33)	0.32 (0.12)	1.96 (0.20)	2.44 (2.01 | 2.87) ∗	−1.64 (−1.21 | −2.86) ∗	0.80 (0.37 | 1.23) ∗

Averages are presented with standard errors in parentheses. Differences between averages, as calculated by Tukey’s HSD, are also shown for each comparison with upper and lower 95% confidence intervals. Asterisks indicate significant differences between averages at a level of p < 0.05.

## Discussion

Here, we contribute to the growing evidence that artificial light influences both the functional and taxonomic structure of communities. Specifically, we provide experimental evidence that the introduction of artificial light increases, or locally clusters, arthropod communities. However, this increase in biomass was not uniform among taxa and trophic guilds. We illustrate that not all arthropods show the same affinity toward artificial light, and some taxa and trophic guilds seem to avoid light or decrease in abundance in the presence of light. While parasites, predators, and scavengers show up to a 4-fold increase in abundance, herbivore abundance decreased when light is present. These shifts in predator and scavenger abundance match previously observed results (see Davies et al.^22^). Our results run counter to observations seen with changing natural light levels where increased natural light decreases predator activity,^14^^,^^35^ thus reaffirming that the *night-light niche* is a novel behavior in response to artificial light that can influence the patterns of nocturnal predators. These results also run counter to the findings of Davies et al. which suggest that artificial lights may shift diurnal arthropod communities.^36^ This suggests that although artificial light may impact diurnal communities, these impacts do not immediately occur upon the introduction of ALAN and may be the result of the prolonged introduction of artificial light to an area.

If artificial lights increase predation pressure around them, they may act as an ecological sink for many arthropod species. Many nocturnal species avoid high levels of light as they are associated with higher predation risks (e.g.,^37^), but our results indicate that the introduction of light drew in individuals and increased the abundance and diversity of communities under lights. Further, while most families returned to pre-light levels once the light was removed, Tingidae did not, indicating different potential lags in how groups of arthropods respond to light.

Currently, cities host rich assemblages of native and non-native species and are of growing importance to biodiversity conservation goals.^38^^,^^39^ However, it is not always clear what species from the regional pool will be able to adapt and survive in urban environments.^40^^,^^41^ As the growth of many current and future cities poses threats to biodiversity hotspots, many researchers have called for a greater understanding of the mechanisms that act as filters and shape urban biodiversity.^31^^,^^42^ Our results provide mechanistic evidence of light pollution acting as an ecological filter to reduce herbivore populations and may speak to a larger pattern of arthropod decline seen worldwide.^43^^,^^44^ Currently, conservationists look to greenspaces embedded in cities as potential solutions to maintaining urban biodiversity. While urban greenspaces can provide habitat for many species, city planners often increase lighting in parks to increase human safety and usability.^45^ This creates a conflict that threatens biodiversity as artificial lighting often acts as an ecological trap for arthropods, thus negating the ecological value of protected open spaces.^46^

Mortality associated with artificial light could act as a strong selective pressure for species to avoid artificial lighting.^44^^,^^47^ With most cities in a near-constant state of brightness, species that live in urban and peri-urban areas already show forms of light-avoiding behavior, but no such adaptation has been seen for rural species.^48^^,^^49^ It is unlikely that species in environments that are currently unaffected by ALAN will be able to adapt at a rate that matches the rapid spread of ALAN to novel environments.^9^ As a result, the rapid introduction of ALAN, especially in biodiversity hotspots, poses a great threat to species as even a small amount of light has been shown to cause ecological shifts.^50^ As cities and ALAN expands, species that are unable to avoid potential ecological traps or increased rates of predation are likely to be lost from the community, suggesting that further investigation into the influence of artificial light biodiversity is needed. Research like ours can contribute to more complex community filter models that will provide a comprehensive understanding of urban/anthropogenic impacts on ecosystem function and biodiversity. Just as we consider novel strategies to combat the impacts of climate change (e.g., assisted migration plans) and increased pollution (e.g., the bioengineering of plastic-consuming microbes), so must we consider inventive solutions to mitigate the impacts of light pollution. Understanding the influence of artificial light on communities and the resulting ecological interactions that may occur is a critical first step in identifying appropriate actions to take for the future planning and policy associated with artificial light.

### Limitations of the study

There are two main limitations of this study. The first limitation is the research was conducted at a single site, and thus, the results may be limited in global applications. Specifically, the arthropod community studied was that of the Northeastern United States, which is a temperate environment. Due to high levels of variability in the arthropod community over the season, the results of this work are also most applicable to the arthropod community present in the late fall. A second limitation of the study is that not all individual arthropods were identified at the species level. Several groups of arthropods, such as Coleoptera (and specifically carabids), have extensive documentation that allows for precise levels of documentation, while other groups are understudied and less readily identifiable beyond the family level.^51^^,^^52^ A final limitation of this study is the limited time over which it is conducted. However, due to the rapid turnover in arthropod communities, we wanted to ensure that the community we sampled at the start of the study had the potential to persist until the end of the study.

## STAR★Methods

### Key resources table

**Table:** 

REAGENT or RESOURE	SOURCE	IDENTIFIER
**Software and algorithms**		

R	R Core Team 2022^53^	https://www.R-project.org/
Package Vegan	Oksanen 2018^54^	https://cran.r-project.org/web/packages/vegan/index.html
Package pairwiseADONIS	Arbizu^55^	https://rdrr.io/github/Jtrachsel/funfuns/man/pairwise.adonis.html

### Resource availability

#### Lead contact

For further information and requests for resources and data, please contact the lead contact, Jeffrey A. Brown (brownjeffrey@lasalle.edu or jeff.alexander.brown@gmail.com)

#### Materials availability

No materials were generated as a result of this work.

### Methods details

#### Study site

We conducted our study at Rutgers University’s Hutcheson Memorial Forest Center (HMFC), an ecological preserve and research facility located in Franklin Township, New Jersey. HMFC, which consists of nearly 200 ha of protected old-growth oak-hickory forest, provides an ideal area to study light pollution because it is undeveloped and closed to the public. These two factors significantly mitigate the amount of ecological light pollution in the area and prevent human disturbances from influencing our data. The study was conducted along a pathway, 10m wide, that was created during the installation of a deer exclosure fence during the summer of 2015. Following the fence construction, plants naturally recolonized the path so that at the time of study it was dominated by a patchwork of grasses and herbs. The pathway was semi-regularly mowed 5–6 times per summer to a height of approximately 2 in. No mowing occurred during our study and the start of our sampling occurred seven days after the previous mowing.

#### Experimental design

We conducted a pitfall trap experiment for fifteen days in August 2016 (August 7^th^ -August 22^nd^). Pitfall traps were comprised of two plastic party cups, one inside the other, buried flush with the ground. This configuration resulted in a 12 cm deep pitfall trap with a 9.5 cm diameter opening. The double cup design allowed us to easily remove and replace traps without disturbing the soil. Approximately 1/8^th^ of the pitfall trap was filled with water and several dashes of unscented soap to reduce surface tension and ensure any arthropod that fell within the pitfall trap would remain trapped. A total of 20 traps were set with each trap placed a minimum of 15 m apart (Figure 1). An LED landscaping light was suspended 2.5 m above 10 of the traps (experimental traps), while the other 10 traps without suspended lights acted as controls. The lights were placed at a height of 2.5 m so that the light that reached the ground was within the suggested illumination levels for streetlights as recommended by the United States Department of Transportation.^56^ The lights produced a maximum lux of 1,800 lumens, had a color temperature of 3,000 K, and a wavelength of approximately 550 nm. To measure light levels, a photometer (ExTech LT45 LED Light Meter) that samples at 2.5 times per second with a sensitivity of 0.01 Lux and peak sensitivity at 550 nm was used. The light level was measured at ground level by placing the sensor of the photometer next to the lip of the pitfall traps. The control traps were interspersed evenly between the experimental traps at least 15 m from the nearest experimental trap.

During each day of the experiment, we sampled twice a day 20 min before civil dawn and 20 min after civil dusk. We selected civil dawn and dusk (when the sun is 6° below the horizon) to sample as this is when streetlights and house lights are typically turned on. Collecting samples twice a day allowed us to distinguish between diurnal and nocturnal communities.

For the first 5 days of the experiment (days 1–5), lights remained off while sampling occurred. For the next 5 days (days 6–10), the light was turned on at night after samples were collected at dusk and turned off before samples were collected at dawn (Figure 1). For the final 5 days (days 11–15), the lights remained off and sampling continued as normal. All samples were placed in ethanol and stored in a freezer to ensure they were well preserved until identification and sorting.

#### Identification

We identified all caught individuals to the family level. Commonly occurring taxa were identified to the genus or species level. We also categorized caught individuals into flying or non-flying groups and placed them into a functional guild based on dietary information. As pitfall traps are not ideal for capturing flying arthropods, we did not include these species in our analysis. The removal of flying arthropods also ensures that traps were far enough apart to be considered independent for the sake of analysis. Additionally, all Collembola were removed from our analysis as they were often not identifiable beyond order. The number of Collembola was also an order of magnitude larger than all other taxonomic groups combined.

### Quantification and statistical analysis

We compared the average species abundance for each day and night (dawn to dusk and dusk to dawn) over the three conditions (pre-light, during-light, post-light) for all experimental pitfall traps and all control traps. Specifically, we conducted a mixed-effects two-way ANOVA with the number of arthropods caught per site, per day as the dependent variable (See Figure 2 and Table 2). Our independent variables were the period (pre-light, during-light, or post-light) and the control treatment (with or without a light). We used the site as a random effect to account for unmeasured differences in the arthropod communities at a single site. We adjusted p-values using the pairwise test function in R with the Bonferroni method to account for the multiple comparisons made within the ANOVA (i.e., three time periods and the control vs. experimental group) The p-value cut-off for this method was 0.05 (See Figure 2 and Table 2). We also used Tukey’s Honest Significant Difference Test (HSD) to compare differences between groupings of our ANOVA to better understand what differences may drive any significance seen from the ANOVA (See Figure 2 and Table 2). All Analysis was conducted in program R version 4.0.4.^53^ We conducted the remaining analysis only on samples collected from experimental pitfall traps. Using the same methods, we compared the average abundance of each guild per night during the three conditions (See Figure 3 and Table 3).

Following this analysis, we compared the community composition of individuals we collected each day and night. Communities were represented by the abundance of each family collected and compared using a principal component analysis (PCA) with package Vegan (Oksanen 2019). We assigned all communities to a cluster based on when the community was collected (i.e., pre-light exposure, during-light exposure, post-light exposure). We compared whether clusters were distinct using permutational multivariate ANOVA with PERMANOVA and tested for differences in dispersion using betadisper, which are both in package Vegan (Oksanen 2018). Direct comparisons between clusters were conducted using pairwise PERMANOVA with package pairwiseAdonis.^55^ Comparing differences between clusters with PERMANOVA allows us to determine if the arthropod communities are compositionally different (See Figure 4 and Tables S1 and S2). Testing the dispersion of the clusters allows us to see whether or not one community is more or less taxonomically diverse (higher dispersion indicating higher taxonomic diversity within a cluster; See Table S2). Finally, we tested the fit of each community in its assigned cluster using hierarchical clustering and checked the fit of each cluster with package pvclust to ensure the clusters accurately represent the data and that observed differences are not an artifact of how we grouped data.^57^ All comparisons were made at the p-value of 0.05. The final step of analysis involved using the results of this PCA to assess the weighting of each family in terms of how important it was to distinguish a particular community composition. All families with over 0.1 or under −0.1 wt were selected to investigate how their abundance changed across the three time periods. The total abundance per site for each period was compared using following the same methods used to compare treatments and guilds mentioned above (See Figure 5 and Table 4).

## Data Availability

• For family level data or overall abundance data, please contact Jeffery A. Brown at either brownjeffrey@lasalle.edu or jeff.alexander.brown@gmail.com. • We did not create any new code for this manuscript. • Additional information: Any additional information required to reanalyze the data reported in this paper is available from the lead contact – jeff.alexander.brown@gmail.com – upon request.
