# Supplementary material for: Introduction of artificial light at night increases the abundance of predators, scavengers, and parasites in arthropod communities
Source: iScience. 2023 Feb 14;26(3):106203. doi: 10.1016/j.isci.2023.106203 (PMC9982679; doi:10.1016/j.isci.2023.106203)
Supplement: Document S1. Tables S1 and S2 [file mmc1.pdf]

iScience, Volume *26*

Supplemental information

Introduction of artificial light at night increases the abundance of predators, scavengers, and parasites in arthropod communities

Jeffrey A. Brown, Julie L. Lockwood, Max R. Piana, and Caroline Beardsley

Title: Supplemental Table 1 – Results from PERMANOVA related to Figure 4

Supplemental Table 1: Results from pairwise PERMANOVA comparing community composition between experimental sites. Sum of squares shown for each comparison along. Comparisons that are significantly different at the $p < 0.05$ level are denoted by an asterisk. Related to Figure 4 and results of PCA.

	Pre-Light Experimental	During-Light Experimental	Post-Light Experimental	Pre-Light Control	During-Light Control	Post -Light Control
Pre-Light Experimental	0	5.93 *	1.40	0.19	0..37	0.28
During-Light Experimental	-	0	7.14 *	6.23 *	6.28 *	5.86 *
Post-Light Experimental	-	-	0	0.61	0.48	0.55
Pre-Light Control	-	-	-	0	0.09	0.012
During-Light Control	-	-	-	-	0	0.08
Post-Light Control	-	-	-	-	-	0

Title: Supplemental Table 2 – Results from TukeyHSD related to Figure 4

Supplemental Table 2: Results from TukeyHSD comparing dispersion as calculated by the betadisper function. Difference between group dispersion is shown. Astrisks indicate the difference is significant at the level of $p < 0.05$. Related to Figure 4 and results of PCA.

	Pre-Light Experimental	During-Light Experimental	Post-Light Experimental	Pre-Light Control	During-Light Control	Post -Light Control
Pre-Light Experimental	0	-2.67 *	0.32	0.43	-0.27	-0.28
During-Light Experimental	-	0	2.99 *	-2.24 *	2.48 *	-2.38 *
Post-Light Experimental	-	-	0	0.47	0.02	0.75
Pre-Light Control	-	-	-	0	0.16	0.15
During-Light Control	-	-	-	-	0	0.06
Post-Light Control	-	-	-	-	-	0
